# Treatment of Coronary Bifurcation Stenoses Using the DK Mini Crush Technique—Analysis of Resources and Technical Success

**DOI:** 10.1002/ccd.70088

**Published:** 2025-08-13

**Authors:** Florian Weidinger, Stephan Achenbach, Johannes Michael Altstidl, Merve Günes‐Altan, Maximilian Moshage, Monique Tröbs, Mohamed Marwan, Luise Gaede

**Affiliations:** ^1^ Department of Medicine 2 – Cardiology and Angiology Friedrich‐Alexander‐Universität Erlangen‐Nürnberg Erlangen Germany

**Keywords:** bifurcation, DKMC, DK crush, double‐kissing crush, 2‐stent strategy

## Abstract

**Background:**

The DK‐mini‐Crush (DKMC) technique, an established strategy to treat coronary bifurcation stenoses, is known to be complex, with possible strain on time and resources.

**Aims:**

To analyze predictors of technical failure as well as time and resourced required for each procedural step of th DKMC technique.

**Methods and Results:**

We prospectively enrolled 105 patients scheduled for coronary revascularization using the DKMC technique. Technical failure—defined as the inability to complete all mandatory procedural steps or any switch to other bifurcation stent deployment techniques—occurred in 10% (11/105) of the patients, most commonly due to inability to correctly place the side branch (SB) stent (3/11) or the inability to perform the 1st kissing balloon dilatation (KBD; 4/11). Even procedures with technical success required material that exceeded the base requirement in 83% of cases and the average procedure time was 47:24 min (IQR 39:00–57:09). The most time and resource‐intense steps were the placement of the SB balloon for the 1st (additional material 48%; 01:38 min [00:35–03:23]) and the 2nd KBD (additional material 58%; 02:10 min [IQR 01:00–04:18]). Performance of a 1st proximal optimization technique (POT)—following crush of the SB stent—was associated with reduced overall need for additional material (OR 0.164 [95% CI 0.046–0.588], *p* = 0.006).

**Conclusion:**

Technical failure occurs in approximately 10% of DKMC procedures and is mainly due to challenges in SB stent placement or inability to perform the 1st KBD. Importantly, a 1st POT is associated with reduced resource utilization and might help to simplify this technique.

## 1 Background

The Double‐Kissing‐Crush (DK‐Crush) technique and its modifications such as the DK‐Mini‐Crush (DKMC) technique are established a priori 2‐stent strategies for the interventional treatment of bifurcation lesions. Large randomized trials have demonstrated that the DK‐Crush technique shows a lower rate of target lesion failure in true bifurcations particularly in the left main (LM) when compared to provisional stenting or other two‐stent techniques [1, 2, 3]. Consequently, it is the only 2‐stent strategy for the treatment of bifurcation lesions that is specifically mentioned in the European Society of Cardiology (ESC) guidelines [4].

However, any clinical advantage of the DK Crush techniques can only be expected in the absence of a stent gap and if the procedure is completed with a final kissing balloon dilatation (KBD) and proximal optimization technique (POT) [5]. Among the many steps which are necessary to achieve this goal, these techniques harbor specific challenges: these include the need for a very precise placement of the side branch (SB) stent with only minimal protrusion into the main branch (MB) [3, 6], the rewiring of the SB twice through crushed stent layers as well as advancing the balloons for both KBDs into the SB, again through several stent layers [7]. Thus, DK Crush techniques are generally considered to be complex, time‐consuming and resource‐intensive [7]—even though published data report very high technical success rates and acceptable procedure duration [1, 2, 3, 8, 9].

The DK Mini Crush (DKMC) technique is one of numerous modifications of the classic DK crush technique, that has become established in everyday clinical practice in recent years. The difference between the techniques lies in the placement of the SB stent: While the classic DK Crush with a 4‐ to 5 mm protrusion of the SB stent results in a relatively large volume of crushed stent in the MB, the DK Mini Crush (DKMC) technique, which aims for only 2 mm protrusion, leaves less stent material in the MB, thus simplifying the entire procedure and raising rates of final KBD up to 98% and on this way possibly long‐term results [10].

The aim of our study was to identify predictors for technical success and failure in lesions scheduled to be treated with the DKMC technique as well as the identification of those procedural steps that are prone to contribute to failure or that are particularly time‐ or resource‐intensive.

## 2 Materials and Methods

Consecutive patients with coronary bifurcation lesions that were planned to undergo percutaneous coronary intervention (PCI) using the DKMC technique performed by an experienced operator were prospectively and consecutively enrolled. An experienced operator was defined as having performed at least 100 cases with the DKMC technique before the study. The decision to perform DKMC was made by the operator depending on anatomical, morphological and patient‐related criteria.

Baseline patient and lesion characteristics as well as interventional details were recorded. The angle of the bifurcation was measured in the projection in which the angle was greatest using a digital triangle. The total procedure time as well as the fluoroscopy time, the radiation dose and the amount of contrast medium needed were recorded, with the start of the procedure defined as the start of the 1st wiring of either branch. The end of the procedure was defined as the completion of the final POT balloon dilatation (the completion of bifurcation treatment). Further stent implantation in the target vessel was allowed following or before bifurcation treatment, but was not considered part of the procedure for the protocol, so that it had no influence on the recorded times and material.

**FIGURE 1:**
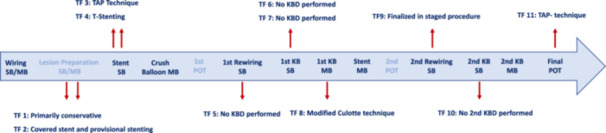
Steps of the DKMC technique (dark blue mandatory steps, light blue not‐mandatory steps), showing the time point and the final technique for each procedure. KBD‐kissing balloon dilatation; MB‐main branch; POT‐proximal optimization technique; SB‐side branch; TF‐technical failure; TAP‐t and protrusion. [Color figure can be viewed at wileyonlinelibrary.com]

Procedural success was defined as angiographic success with an angiographic residual stenosis < 10% and thrombolysis in myocardial infarction (TIMI) flow grade III in both vessels. Technical success was defined as the ability to complete all mandatory steps of the protocol (Figure 1) and no need to switch to other established stent techniques. For all patients with technical failure, the procedural step at which the failure occurred, the detailed reason for the failure as well as the final technique in which the procedure was completed, were recorded. The protocol was stopped at the moment of technical failure. Only patients without technical failure—that is, patients with technical success—were included in the detailed analysis of time and resource consumption to exclude incomplete datasets and to carry out valid statistical comparisons.

### 2.1 Definition of the Recorded Steps

The protocol included the following partly mandatory and partly optional steps according to the recommendations of the European Bifurcation Club recommendation [7]:

Wiring of the SB, wiring of the MB, lesion preparation SB, lesion preparation MB, stent placement SB, crush‐balloon placement MB, 1st POT, 1st rewiring SB, placing of the balloon for the 1st KBD in the SB, placing of the balloon for the 1st KBD in the MB, stent placement in the MB, 2nd POT, 2nd rewiring of the SB, placing of the balloon for the 2nd KBD in the SB, placing of balloon for the 2nd KBD in the MB, 3rd (final) POT. With the exception of lesion preparation in either branch as well as the 1st and 2nd POT all steps were defined as mandatory (Figure 1).

### 2.2 Detailed Time and Material Analysis

Detailed time and material analysis was made for SB wiring, MB wiring, stent placement into the SB, crush‐balloon placement into the MB, 1st rewiring SB, 1st KB placement SB, 1st KB placement MB, stent placement MB, 2nd rewiring SB, 2nd KB placement SB, 2nd KB placement MB. For all other steps (lesion preparation, 1st, 2nd and 3rd [final] POT), the only parameter recorded was whether they were carried out or not.

Detailed time analysis comprised the duration of each step measured in seconds. For all steps describing wiring, the start time was defined when the wire was introduced in the guiding catheter, completion of wire placement and advancing to the next step of the procedure represented the end time. For all steps describing the placement of balloons or stents, the time recording started when the operator began to place the intended device or any other device to facilitate the procedure—such as a smaller balloon—onto the coronary guidewire and stopped after inflating the intended balloon or stent. The operator gave the start and stop signal for each individual step. The time itself was transferred from the display of the coronary angiography system to the second.

Resource analysis recorded any material (e.g., coronary guidewires or stents) in addition to basic requirements. The basic material comprised two wires, any material for initial lesion preparation at the discretion of the operator including balloons or debulking devices, two drug‐eluting stents (DES), two noncompliant balloons for KBD (one each for the side branch and one for the main branch), and one noncompliant balloon for the POT maneuvers. Accordingly, the need for additional material was defined if more than two wires throughout the whole procedure, more than two balloons for the two KBD and more than one balloon for the POT were required.

### 2.3 Statistical Analysis

Statistical analysis was performed using the IBM SPSS statistics program (version: 28.0.0.0 (190)). The data was checked for normal distribution using the Kolmogorov‐Smirnov test. In the case of normally distributed data, mean values with standard deviation are provided. If data were not normally distributed median values and interquartile range (IQR) are provided. Proportionate values were shown in per‐cent together with the absolute number (n) where appropriate. The chi‐square test was used to compare categorial variables; Fisher´s exact test was performed for group sizes ≤ 5. For comparisons of continuous data in independent samples, variance homogeneity was checked using Levene tests and differences between the two distributions were analyzed using a *t*‐test. In the case of variance heterogeneity, a Man‐Whitney U test was applied. The odds ratio (OR) with a 95% confidence interval (CI) was calculated for the variables that showed a significant difference between the group that required additional material and the group that did not require additional material. Regression analysis was used to examine the association between variables. For all analyses, a *p* value < 0.05 was considered to indicate statistical significance.

The study was in accordance with a positive vote by the local ethic committee (Vote number 94_21B).

## 3 Results

### 3.1 Patient Demographics and Procedural Characteristics

Between December 2018 and June 2024, 105 consecutive patients were enrolled in this study. Median age of the study population was 69 years (IQR 60–77) and most patients (74%) were male. Most cases involved the left anterior descending artery (LAD) as a MB (69%, 73/105), followed by the LM (20%; 21/105). All but one lesion were true bifurcations involving the MB and SB, most (78%) were classified as Medina 1‐1‐1. Baseline and procedural characteristics are shown in Table 1. Procedural success was achieved in 99%. With regard to the non‐mandatory steps of the technique, 85% of procedures involved lesion preparation of the MB and 88% of the SB. The 1st POT—following crush of the SB stent—was performed in 14%, the 2nd POT—after implantation of the MB stent—in 67% of the procedures. All data are shown in Table 1.

**TABLE 1:** Clinical and procedural characteristics of patients with and without technical success.

Clinical and procedural characteristics	All patients (*n* = 105)	Technical success (*n* = 94)	Technical Failure (*n* = 11)	*p* value
Age (Median; IQR)	69 (60–77)	69 (60–76)	75 (58–82)	0.414
Male	74% (78)	72% (68)	91% (10)	0.281
BMI (kg/m^2^)	28.06 (± 5.45)	27.97 (± 5.59)	28.83 (± 4.16)	0.323
Main branch				0.491
LM	20% (21)	21% (20)	9% (1)	
LAD	69% (73)	69% (65)	73% (8)	
LCX	9% (9)	7% (7)	18% (2)	
RCA	2% (2)	2% (2)	0% (0)	
Vessel Reference diameter (mm)*				
P‐MB	3.8 (± 0.5)	3.8 (± 0,5)	3.6 (± 0.4)	0.135
D‐MB	3.2 (± 0.4)	3.2 (± 0,4)	3.0 (± 0.3)	0.186
SB	2.7 (± 0.4)	2.7 (± 0,4)	2.6 (± 0.3)	0.529
Grade of stenosis (mean %)*				
P‐MB	61 (± 28)	59 (± 29)	77 (± 16)	0.071
D‐MB	75 (± 19)	75 (± 19)	80 (± 14)	0.360
SB	78 (± 15)	77 (± 14)	85 (± 17)	0.107
Medina‐ Classification (%; *n* = 104)				0.789
1‐1‐1	78% (81)	77% (72)	90% (9)	
1‐1‐0	1% (1)	1% (1)	0% (0)	
1‐0‐1	3% (3)	3% (3)	0% (0)	
0‐1‐1	18% (19)	19% (18)	1% (1)	
Indication for intervention				0.853
CCS	85% (89)	84% (79)	91% (10)	
ACS	15% (16)	16% (15)	9% (1)	
Radial access	79% (83)	79% (74)	82% (9)	1.000
Guiding catheter				0.455
6 F	80% (84)	79% (74)	91% (10)	
7 F	20% (21)	21% (20)	9% (1)	
Angle < 70°	77% (81)	78% (73)	73% (8)	0.662
Lesionpreparation done				
MB	85% (89)	84% (79)	91% (10/11)	0.195
SB	88% (92)	89% (84)	73% (8/11)	0.324
1st POT done	14% (14)	14% (13)	14% (1/7)	0.660
2nd POT done	67% (64)	68% (63)	33% (1/3)	0.548
Procedural Success	99% (105)	100% (94)	91% (10/11)	0.105

*Note:* Values are mean (+/− SD), median (IQR) or n (%).

Abbreviations: ACS, Acute coronary syndrome; BMI, Body Mass Index; CCS, chronic coronary syndrome; D‐MB, distal main branch; F, French; LM, left main; LAD, left anterior descending; LCX, left circumflex; P‐MB, proximal main branch; POT, proximal optimization technique; RCA, right coronary artery; SB, side branch.

### 3.2 Technical Success

Technical success according to our definition was achieved in 90% (94/105).

A detailed description of the 11 procedures with technical failure containing, including the reason and the procedural step of failure as well as the bailout strategy is provided in Figure 1, with further information in thein supporting material (Figure 1; Table S1):

Briefly, the procedure failed in two cases during MB preparation, in two cases during placement of the SB stent, in 1 case during the 1st rewiring of the SB, in two cases during the balloon placement for the 1st KBD in the SB, in one case during balloon placement for the 1st KBD in the MB, in one case during the balloon placement for the 2nd KBD in the SB and in one case during the final POT. One procedure had to be interrupted during 2nd rewiring of the SB due to acute stent thrombosis with consecutive hemodynamic deterioration, cardiopulmonary resuscitation and implantation of a left ventricular assist device. Besides this failed case, all cases showed final procedural success with TIMI III flow at the end of the procedure and a residual stenosis < 10%.

In summary, technical failure was due to difficulties in SB stent placement in 27% (3/11), due to difficulties either during the 1st (4/11) or the 2nd KBD (1/11) in 46% or due to procedural complications independent of the DKMC technique in 27% (3/11) of the procedures in which the DKMC technique could not be completed as intended.

Overall, this resulted in a change to other stent techniques in 46% (5/11) of the cases: two cases were finished in the TAP technique, one in T‐Stenting, one in Culotte and one without side branch stent. In 45% (5/11) of the cases the DKMC technique was carried out, but not all mandatory steps of the DKMC technique could be performed: In three cases, neither the 1st nor the 2nd KBD was performed, in another case only the 2nd KBD failed because no balloon could be brought into the SB. One case was completed in a staged procedure. Finally, one lesion was treated conservatively as lesion preparation failed.

Despite a numeric trend toward technical failure occurring more often in older and male patients and in the presence of higher grade stenoses in all vessel sections related to the bifurcation, no statistically significant difference in patient characteristics, lesion characteristics (e.g., location, vessel size) or periprocedural characteristics (e.g., guiding size, access route) between procedures that were successfully completed and those with technical failure was observed (Table 1).

### 3.3 Procedural Times

**FIGURE 2:**
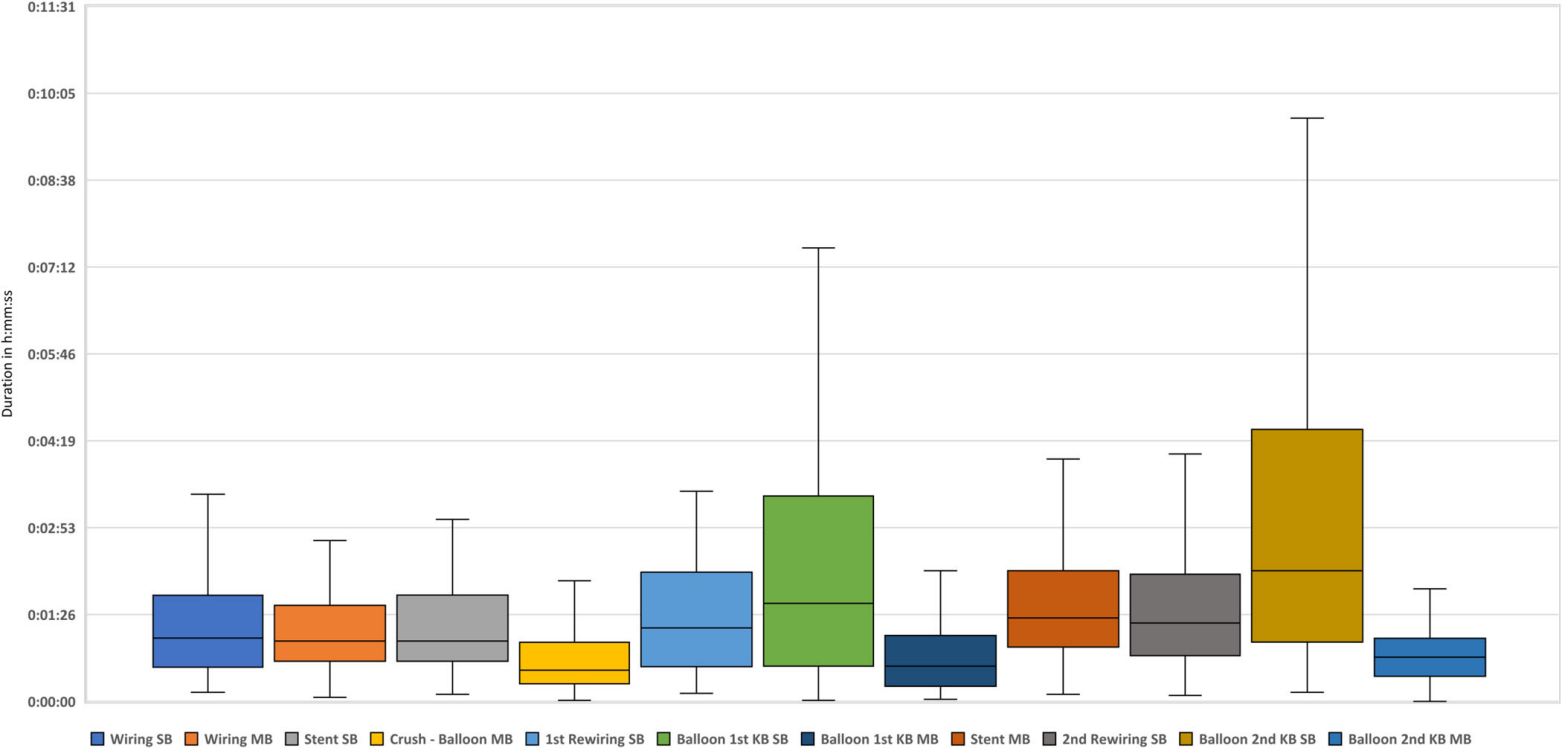
Median times of the single steps of the DKMC‐technique. [Color figure can be viewed at wileyonlinelibrary.com]

The median overall procedural duration for successfully completed DKMC procedures (*n* = 94) was 47:24 min (IQR 39:00–57:09), the mean fluoroscopy time 15:51 min (± 08:45), the mean area dose product 4843 µGym² (± 3991) and the mean amount of contrast agent 124 ml (± 55). The most time‐consuming step was balloon placement for the 2nd KBD in the SB with a median duration of 02:11 min (IQR 01:00–04:43). The fastest step was the placement of the crush‐balloon in the MB with a median duration of 00:31 min (IQR 00:19–00:58). The times of each individual step of the DKMC method are shown in detail in Figure 2 and Table 2.

**TABLE 2:** Median duration of each step and overall procedure time as well as men radiation, fluoroscopy and contrast medium amount of the DKMC technique stratified into patients with and without the need for any additional material.

Procedural steps	Overall	Without any additional material (*n* = 16)	Need of additional material (*n* = 78)	*p* value
Wiring SB	01:03 (00:35–01:45)	00:42 (00:31–01:34)	01:07 (00:43–01:47)	0.363
Wiring MB	01:00 (00:40–01:33)	00:45 (00:29–01:20)	01:00 (00:42–01:37)	0.239
Stent SB	01:00 (00:40–01:45)	01:02 (00:41–01:23)	01:00 (00:40–01:53)	0.402
Crush ‐ Balloon MB	00:31 (00:19–00:58)	00:30 (00:20–00:50)	00:34 (00:17–00:58)	0.956
1st Rewiring SB	01:13 (00:35–02:07)	01:02 (00:35–02:04)	01:15 (00:36–02:07)	0.439
Balloon 1st KB SB	01:38 (00:35–03:23)	00:48 (00:29–01:29)	01:54 (00:41–03:43)	0.069
Balloon 1st KB MB	00:35 (00:15–01:05)	00:36 (00:23–01:01)	00:35 (00:14–01:06)	0.560
Stent MB	01:23 (00:54–02:10)	01:29 (00:39–02:17)	01:23 (00:55–01:58)	0.572
2nd Rewiring SB	01:18 (00:47–02:05)	01:04 (00:53–02:23)	01:20 (00:45–02:01)	0.743
Balloon 2nd KB SB	02:10 (01:00–04:18)	00:59 (00:21–01:20)	02:18 (01:28–04:50)	0.052
Balloon 2nd KB MB	00:44 (00:25– 01:03)	00:47 (00:25–01:13)	00:44 (00:25–01:03)	0.506
Procedural time in min.	47:24 (39:00–57:09)	40:10 (30:20–44:51)	49:15 (40:59–57:46)	0.084
DAP in µGym²	4843 (± 3991)	3929 (± 2509)	5019 (± 4206)	0.336
Fluoroscopy in s	951 (± 534)	780 (± 475)	984 (± 543)	0.169
Contrast medium (mL)	124 (± 55)	128 (± 55)	123 (± 55)	0.733

*Note*: Values are mean (+/− SD), median (IQR) or n (%).

Abbreviations: DAP, Dose area product; KBD, kissing balloon dilatation; MB, main branch; SB, side branch.

### 3.4 Resources

**FIGURE 3:**
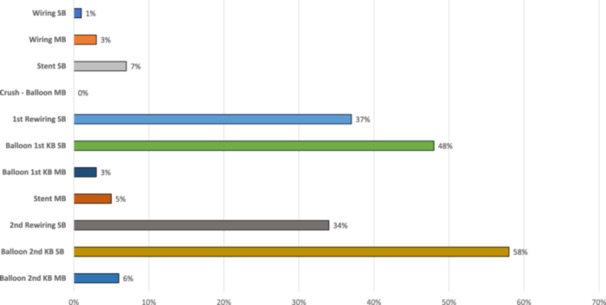
Percentage of procedures in which additional material was needed in each step of DKMC. [Color figure can be viewed at wileyonlinelibrary.com]

Overall, 83% (78/94) of the successful procedures required additional material. Additional wires were needed in 56% (*n* = 53) and additional balloons in 72% (*n* = 68) of the procedures. The frequencies in which additional material was used depending on each procedural step, are shown in Figure 3 and Table 3. The additional material was mainly used for the 2nd rewiring of the SB, which required additional wires in 34% of cases and for the placement of the SB balloon for the 2nd KBD, which required additional balloons in 58% of the cases. The balloon placement in the side branch for the 2nd KBD not only required the most additional material, but this step also used the most material with 38 cases in which one balloon was used, eight cases in which two balloons were used, four cases in which three balloons were used, two cases in which four balloons were used and three cases in which even five balloons were used. The exact details of the amount of additional material for each step are listed in Table S2.

**TABLE 3:** Additional material for each procedural step and time analysis depending on the need for additional material for each single step.

Procedural Step	Additional material needed (%, n)	Time for cases without additional material	Time for cases with additional material	*p* value
Wiring SB	1% (1)	01:02 (00:33–01:45)	18:28	**< 0.001**
Wiring MB	3% (3)	01:00 (00:40–01:31)	02:40 (00:23–04:50)	0.105
Stent SB	7% (7)	01:00 (00:39–01:40)	01:55 (01:26–02:57)	0.081
Crush ‐ Balloon MB	0% (0)	00:31 (00:19–00:58)	—	—
1st Rewiring SB	37% (35)	01:07 (00:30–01:49)	01:45 (00:51–02:40)	0.052
Balloon 1st KB SB	48% (45)	00:36 (00:24–01:12)	03:24 (02:25–06:20)	**< 0.001**
Balloon 1st KB MB	3% (3)	00:35 (00:15–01:05)	00:51 (00:08–03:00)	0.260
Stent MB	5% (5)	01:21 (00:47–01:58)	04:01 (03:20–11:17)	0.153
Balloon 2nd Rewiring SB	34% (32)	01:09 (00:48–02:01)	01:27 (00:45–03:46)	0.125
Balloon 2nd KB SB	58% (55)	00:59 (00:22–02:05)	03:12 (01:55–06:25)	**< 0.001**
Balloon 2nd KB MB	6% (6)	00:44 (00:25–01:00)	02:16 (01:05–06:44)	0.237

*Note:* Values are mean (+/− SD), median (IQR) or n (%). The *p*‐values with statistical significance of *p* < 0.05 are marked in bold.

Abbreviations: KBD, kissing balloon dilatation; MB, main branch; SB, side branch.

There was no statistically significant difference between the durations of the individual steps and the overall intervention time, fluoroscopy time and dose area product as well as the amount of contrast medium consumed between patients with the need for any additional material and patients without the need for additional material (Table 2).

Analyzed separately, the time required for balloon placement of the 1st as well as for the 2nd KBD in the SB was significantly prolonged if additional material was needed for this very step. In all other steps the need for additional material did not influence the required time (Table 3).

The individual parameters that were statistically analyzed for their influence on the need of additional material throughout the whole procedure are summarized in Table 4. Apart from the clinical presentation of the patient as an acute coronary syndrome (ACS) in 43% (7/16) without and in 10% (8/78) with the need for additional material (OR 0.147 [95% CI 0.043–0.502], *p* = 0.002), 1st POT, was performed more often (38% vs. 9%, *p* = 0.008; (OR 0.164 [95% CI 0.046–0.588], *p* = 0.006) and the length of the MB stent was shorter (23 mm vs. 27 mm, *p* = 0.038) in procedures without additional material. No other statistically significant differences could be seen between the two groups.

**TABLE 4:** Clinical and procedural characteristics of patients with and without the need for any additional material.

Clinical and procedural characteristics	All patients (*n* = 94)	Intervention without additional material (*n* = 16)	Intervention with additional material (*n* = 78)	*p* value
Age (median, IQR)	69 (60–76)	65 (60–89)	70 (60–76)	0.863
Male	72% (68)	63% (10)	74% (58)	0.365
BMI (mean; SD)	27.97 (± 5.59)	27.10 (± 3.45)	28.15 (± 5.94)	0.497
Main branch				0.902
LM	21% (20)	25% (4)	20% (16)	
LAD	69% (65)	69% (11)	69% (54)	
LCX	7% (7)	6% (1)	8% (6)	
RCA	2% (2)	0% (0)	3% (2)	
Vessel Reference diameter (mm)*				
P‐MB	3.8 (± 0,5)	3.9 (± 0.8)	3.8 (± 0.5)	0.602
D‐MB	3.2 (± 0,4)	3.2 (± 0.4)	3.2 (± 0.4)	0.914
SB	2.7 (± 0,4)	2.8 (± 0.5)	2.7 (± 0.3)	0.346
Grade of stenosis in % (mean; SD)				
P‐MB	59 (± 29)	48% (± 33)	62% (± 27)	0.094
D‐MB	75 (± 19)	71% (± 21)	75% (± 18)	0.451
SB	77 (± 14)	76% (± 14)	77% (± 15)	0.855
Medina‐ Classification				0.736
1‐1‐1	77% (72)	69% (11)	78% (61)	
0‐1‐1	19% (18)	25% (4)	18% (14)	
1‐1‐0	1% (1)	0% (0)	1% (1)	
1‐0‐1	3% (3)	6% (1)	3% (2)	
Indication for Intervention				**0.001**
CCS	84% (79)	57% (9)	90% (70)	
ACS	16% (15)	43% (7)	10% (8)	
Radial access	79% (74)	81% (13)	78% (61)	1.000
Guiding catheter				0.088
6 F	79% (74)	63% (10)	81% (63)	
7 F	21% (20)	37% (6)	19% (14)	
Bifurcation Angle < 70°	78% (73)	87% (14)	76% (59)	0.510
Lesionpreparation done				
MB	84% (79)	14% (11)	86% (68)	0.125
SB	89% (84)	18% (15)	82% (69)	1.000
SB Stent				
Diameter (mm, *n* = 92)	2,7 (92)	2,8 (16)	2,7 (76)	0,408
Length (mm, *n* = 92)	19 (92)	21 (16)	19 (76)	0,351
Type				0.841
Promus	38% (35)	38% (6)	38% (29)	
Synsiro	34% (32)	31% (5)	35% (27)	
Xience	22% (20)	25% (4)	21% (16)	
Biomatrix Flex	3% (2)	6% (1)	2% (1)	
Other	3% (3)	0% (0)	4% (3)	
MB Stent				
Diameter (mm, *n* = 93)	3,2 (93)	3,3 (16)	3,2 (77)	0.205
Length (mm, *n* = 93)	26 (93)	23 (16)	27 (77)	**0.038**
Type				0.238
Promus	58% (54)	44% (7)	60% (47)	
Synsiro	25% (23)	25% (4)	24% (19)	
Xience	13% (12)	19% (3)	12% (9)	
Biomatrix Flex	2% (2)	6% (1)	2% (1)	
Others	2% (2)	6% (1)	2% (1)	
1st POT done	14% (13)	38% (6)	9% (7)	**0.008**
KBD Diameter				
MB (mm)	3,2	3,3	3,2	0.295
SB (mm)	2,6	2,8	2,6	0.181
2nd POT done	68% (63)	75% (12)	66% (51)	0.357
Final POT balloon diameter (mm)	3,9	4	3,8	0.195

*Note*: Values are mean (+/− SD), median (IQR) or n (%). The *p*‐values with statistical significance of *p* < 0.05 are marked in bold.

Abbreviations: ACS, Acute coronary syndrome; BMI, Body Mass Index; CCS, chronic coronary syndrome; D‐MB, distal main branch; F, French; LM, left main; LAD, left anterior descending; LCX, left circumflex; P‐MB, proximal main branch; POT, proximal optimization technique. RCA, right coronary artery; SB, side branch.

## 4 Discussion

### 4.1 Main Findings

In our all‐comer cohort with coronary bifurcation lesions intended for PCI using the DKMC technique, the DKMC procedure was successfully performed in 90% of patients. The main reasons for technical failure were the unprecise placement of the SB stent or the inability to perform one or both KBDs. If DKMC was performed successfully, the balloon placement for both the 1st and 2nd KBD in the SB consumed the most time and material. The need for additional material significantly prolonged the time required for each of these two steps. Performing a 1st POT—after the placement of the SB stent—was shown to reduce the need for additional material and might facilitate the procedure.

### 4.2 Technical Success of DKMC

The technical success rate of the DKMC technique in our analysis was with 90% relatively high, albeit lower than in prior randomized studies (96%–97%) [1, 2, 3, 9, 11].

Overall, no predictors for technical failure itself could be found in our cohort. However, a closer look at the singular cases can help to explain these lower rates of technical success. For this purpose, the cases can be divided into three groups:

The first group concerns the three cases—around ¼ of the cases with technical failure—in which the technical failure was ultimately attributable to the placement of the SB stent. The placement of the SB stent is the main distinguishing feature between the DKMC technique and the classic DK crush technique. Placement is much more challenging with the DKMC technique, as less protrusion into the MB is desired to minimize the volume of the crushed stent [6]. Our data can show that this not only has advantages, but in some cases also can cause technical failure of the whole procedure. Failures in accurate SB stent positioning resulted in either no protrusion into the MB or gap formation at the proximal SB, necessitating bailout TAP stenting.

The second group‐ presenting around ½ of the cases with technical failure and approximately 5% of all procedures—contains the patients for whom one of the KBDs provoked the technical failure. Performing the final KBD is certainly the Achilles heel of all 2‐stent strategies and especially of any DK crush technique. To understand our cases in more detail, it is important to note that in all but one case, the 1st KBD was the cause. In the evolution of crush techniques, the DK crush technique prevailed over the classic crush and later the DKMC over the DK crush, because a higher rate of the final KBD could be achieved [1, 2, 3, 8, 9, 11, 12]. Due to the constantly decreasing crushed stent mass in the bifurcation area with each further development of the technique, the reported rate of final KBD sometimes even ranges up to 100% nowdays [1, 2, 3, 8, 9, 11, 12]. Compared to these studies, the slightly higher percentage of failure of first and/or final KBD in our cohort might be due to different reasons all explained by the purely observational character of the study: Firstly, patients were not preselected as they are in randomized trial. This usually means that more difficult anatomies, for example, more challenging bifurcation angles or more calcified lesions, are included in the analysis. Secondly, there was no clear specification for the material to be used, for example, the choice of the stent type was up to the interventionalist. Thirdly, no strict study protocol was available: this means that there was a certain degree of freedom in the implementation of the procedure: Some steps, such as the first POT, were not mandatory. Additionally, the position of the side branch wire after rewiring was not systematically evaluated. Both mechanisms might lead to difficulties of balloon crossing of the crushed SB stent for KBD.

The third group after all also representing ¼ of the patients with technical failure were procedures, which failed due to factors independent of the technique itself. Failure of lesion preparation, coronary perforation or acute stent thrombosis are rare challenges and complications, but they still do occur in a certain percentage of PCI cases. These three cases are therefore particularly noteworthy in the overall context of our results, especially as two out of three were after all treated successfully with the DKMC technique, but not in the corresponding procedure in which the protocol was recorded.

In summary, the lower number of technical success of our analysis compared to previous data on DK Crush techniques [1, 2, 3, 9, 11] might be explained by three reasons: (1) the challenge of SB stent positioning posed by the DKMC technique compared to the classic DK crush technique, (2) the observational character of the study (3) a relatively high rate of technical failure due to special individual cases with rare complications.

### 4.3 Overall Procedure Time

Complex 2‐stent strategies are associated with longer procedure times, higher contrast agent consumption, and higher radiation exposure compared to a primary single‐stent strategy [13]. The median intervention time of the DKMC technique in our cohort was 47:24 min (IQR 39:00–57:09). Due to different definitions of procedure duration and, in some cases, additional mandatory interventional steps of the technique our results can best be compared with the DKCRUSH I (46:30 min); and DKCRUSHIII (56:53 min) as well as the ROUTE trial (45:00 min) and lie within the anticipated time range. This confirms the overall time‐consuming aspect of the technique—even if performed by experienced operators and outside of randomized trials. In the future, this may continue to be a reason why DK crush techniques do not prevail compared to other 2‐stent strategies—regardless of the data situation. For example, lately, DK culotte was shown to require only 2/3 of the time of DK Crush [9].

### 4.4 Detailed Time and Material Analysis

One of our main objectives of the study was to analyze the exact time and the need for additional material for each single step of the DKMC technique. To our knowledge, our data are the first to provide results on this topic. Our analysis shows that the time required as well as the need for additional material were particularly high for the steps that took place in the SB. The SB exit angle represents a natural access problem. Additionally, during the DK‐Crush techniques the access to the MB is always ensured, whereas access to the SB is consistently more difficult, beginning with the moment of the crush of the SB stent continuing with the multi‐layer stent struts above the SB ostium after implantation of the MB stent. The further development of DK Crush to DKMC technology should have made things easier here, but our data can still show that the placing of the balloons for the KBD in the SB are the most time and material consuming steps of the DKMC technique: Whereas the need for additional material for these steps also resulted in a prolonged time, the need for additional material in the other single steps showed only a numeric prolongation of the time needed for each very step itself. Surprisingly, the overall need for any additional material throughout the whole procedure did neither result in a longer total procedure time nor in higher radiation, fluoroscopy or contrast medium use. This lack of statistical significance could be explained by our study size, which is nevertheless large for such a detailed analysis.

### 4.5 Prevention of the Need of Additional Material When Performing DKMC

We could identify two predictors that prevent the need for additional material: Firstly, the non‐mandatory 1st POT seems to help in keeping DKMC more straight forward. In the latest publication from the European Bifurcation Club (EBC), which explicitly lists the steps of the DK‐crush technique and can therefore currently be seen as the gold standard recommendation, the 1st POT as such is only mentioned in a subordinate clause [14]. However, benchmark tests have shown that this step is the only way to achieve complete crushing of the SB stent and prevents the risk of distal abluminal rewiring of the SB stent [5]. Additionally, the first POT might result in a better opening of the jailed struts at the SB ostium and a better preparation of the proximal MB segment. All three mechanisms could explain why the lack of the 1st POT does lead to additional material and time consumption. Thus, the execution of the 1st POT should be firmly implemented in the standardized procedure of the DK techniques.

Secondly, patients presenting with ACS needed additional material less often. Due to the different, softer plaque morphology of patients with an ACS, it is conceivable that more complex procedures such as 2 stent strategies in bifurcation lesions are easier to perform in these patients [15].

### 4.6 Limitations of the Study

A larger cohort could provide statistically more robust results, but still the cohort for such detailed analysis is quite big. As the study was a monocentric study, the interventions were performed by only three different investigators with very high expertise in the DKMC technique. This is a bias that is—at least partly—present in any study, especially in the randomized studies on stenting techniques. Another limitation of our analysis is that the focus lied only on the analysis of the detailed interventional data. Additionally, intravascular imaging was used only in a small fraction of procedures (12%). Moreover, even in the rare cases it was not performed systematically for example, for the evaluation of the position of the SB wire after rewiring or for final results. An accurate analysis of this is therefore not feasible. Last, there is no data on long‐term clinical outcome of the patients.

## 5 Conclusion

In an all‐comer cohort, the DKMC technique could be completed with all mandatory steps in 90% of the cases. Technical failure was mainly due to unprecise placement of the SB stent or to the impossibility to perform KBD. Additionally, the technique was shown to be time consuming and additional material was needed in 83% of the cases. The steps that required the most material and time were the placement of the balloons for the KBD in the SB. The standardized performance of the 1st POT after crushing the SB stent could help to reduce the need for additional material in the complex DK Crush techniques.

## Ethics Statement

Ethical approval for this study was obtained from the Ethics Committee of the FAU (Vote 94_21 B).

## Conflicts of Interest

Luise Gaede received speaker honorarium from Abiomed, Abbott Laboratories, AstraZeneca, Boston Scientific, Edwards Lifesciences, Siemens Healthineers, SMT, and Shockwave Medical. J. Michael Altstidl received speaker honorarium from Edwards Lifesciences. Merve Günes‐Altan received travel support from Eli Lilly and Company. Mohamed Marwan received speaker honorarium from Edwards Lifesciences and Siemens Healthineers. Monique Tröbs received speaker honorarium from Abbott Laboratories, Pfizer, and Bristol Myers Squibb, travel support from Abbott Laboratories and Edwards Lifesciences, and participates in the Pfizer Advisory Board. The other authors declare no conflicts of interest.

## Supporting information


**Table S1:** Detailed analysis of each procedure with technical failure ordered according to the time of occurrence in the protocol. **Table S2:** Detailed information about additional material use.
