# Supplementary material for: Treatment of Coronary Bifurcation Stenoses Using the DK Mini Crush Technique—Analysis of Resources and Technical Success
Source: Catheter Cardiovasc Interv. 2025 Aug 13;106(4):2528–38. doi: 10.1002/ccd.70088 (PMC12502013; doi:10.1002/ccd.70088)
Supplement: Supplementary file 1 — Table S1: Detailed analysis of each procedure with technical failure ordered according to the time of occurrence in the protocol. Table S2: Detailed information about additional material use. [file CCD-106-2528-s001.docx]

Table S1: Detailed analysis of each procedure with technical failure ordered according to the time of occurrence in the protocol

| Number  Technical | Procedural Step of technical failure | Reason for Technical Failure | Final Technique/Solution |
| --- | --- | --- | --- |
| TF1 | MB Lesion preparation | No preparation possible | Conservative treatment of the bifurcation, only treatment of another stenosis in this session, staged procedure with stenting **(DKMC)** of the bifurcation after rotational atherectomy |
| TF2 | MB Lesion preparation | Perforation of the MB during lesion preparation | Prolonged balloon inflation in the MB, implantation of covered stent and finalized in single stent technique (**provisional stenting**) without any rewiring of the SB |
| TF3 | SB-Stent implantation | Initially SB-Sent planned for DK crush was implanted to far distally into the SB | 2^nd^ SB Stent implanted in **TAP technique** after MB stent implantation |
| TF4 | SB Stent implantation | DKMC showed not suitable for bifurcation anatomy due to a angle nearly 90° | **T-Stenting** performed |
| TF5 | 1^st^ Rewiring SB | Impossible to rewire SB after the Crush of the SB stent | **Incomplete DKMC**; No KBD (neither 1^st^ nor 2^nd)^ was performed throughout the whole procedure |
| TF6 | Balloon 1^st^ KBD SB | Impossible to place NC-Balloon into SB* | **Incomplete DKMC**; No KBD (neither 1^st^ nor 2^nd)^ was performed throughout the whole procedure |
| TF7 | Balloon 1^st^ KBD SB | Impossible to place NC-Balloon into SB* | **Incomplete DKMC**; No KBD (neither 1^st^ nor 2^nd)^ was performed throughout the whole procedure |
| TF8 | Balloon 1^st^ KBD MB | After rewiring SB and dilatation of the stent struts as well as positioning of the balloon for 1^st^ KBD in the SB, balloon placement for the 1^st^ KBD the MB could not be performed* | Rewiring crushed stent struts of the SB stents to the MB, dilatation, finalization in **modified Culotte** technique. |
| TF9 | 2^nd^ Rewiring SB | Stent thrombosis as well as thromboses within the side branches due to unknown Heparin induced thrombocytopenia of the patient occurred | CPR, placement of a left-ventricular assist device, primarily no 2^nd^ KBD and thus no final POT performed, **DKMC finalized in staged procedure**. |
| TF10 | Balloon 2^nd^ KBD SB | Impossible to place NC-Balloon into SB* | **Incomplete DKMC**; No 2^nd^ KBD Dilatation was performed |
| TF11 | Final POT | At the end of the procedure, final angiography revealed incomplete coverage of the ostial SB | Additional Stent in **TAP-Technique** |

*despite several rewirings, use of mini-balloon and anchor-maneuver

Table S2: Detailed information about additional material use

| Procedural Step | Additional material needed (%, n) | Number of additional wires/balloons for each step | | | | | |
| --- | --- | --- | --- | --- | --- | --- | --- |
|  |  | **0** | **1** | **2** | **3** | **4** | **5** |
| Wiring SB | 1% (1) | **92** | **0** | **0** | **0** | **1** | **0** |
| Wiring MB | 3% (3) | **90** | **3** | **0** | **0** | **0** | **0** |
| Stent SB | 7% (7) | **87** | **6** | **1** | **0** | **0** | **0** |
| Crush - Balloon MB | 0% (0) | **94** | **0** | **0** | **0** | **0** | **0** |
| 1^st^ Rewiring SB | 37% (35) | **58** | **31** | **3** | **1** | **0** | **0** |
| Balloon 1^st^ KB SB | 48% (45) | **49** | **34** | **6** | **3** | **1** | **1** |
| Balloon 1^st^ KB MB | 3% (3) | **90** | **3** | **0** | **0** | **0** | **0** |
| Stent MB | 5% (5) | **83** | **4** | **0** | **0** | **1** | **0** |
| 2^nd^ Rewiring SB | 34% (32) | **61** | **26** | **6** | **0** | **0** | **0** |
| Balloon 2^nd^ KB SB | 58% (55) | **38** | **38** | **8** | **4** | **2** | **3** |
| Balloon 2^nd^ KB MB | 4% (4) | **90** | **2** | **2** | **0** | **0** | **0** |
